# Ethnic Differences in Childhood Nephrotic Syndrome

**DOI:** 10.3389/fped.2016.00039

**Published:** 2016-04-19

**Authors:** Rahul Chanchlani, Rulan S. Parekh

**Affiliations:** ^1^Division of Pediatric Nephrology, Hospital for Sick Children, Toronto, ON, Canada; ^2^Child Health Evaluative Sciences, Research Institute, Hospital for Sick Children, Toronto, ON, Canada; ^3^Division of Pediatric Nephrology, McMaster Children’s Hospital, Hamilton, ON, Canada; ^4^Department of Medicine, Division of Nephrology, University Health Network, Toronto, ON, Canada

**Keywords:** steroid resistant, steroid dependent, minimal change disease, focal segmental glomerulosclerosis, nephrotic syndrome

## Abstract

Nephrotic syndrome is a common glomerular disease in children with significant variability in both incidence and steroid responsiveness among various ethnic groups. The average incidence of nephrotic syndrome is 2–16.9 per 100,000 children worldwide. Understanding the variability by ethnicity may point to potential factors leading to nephrotic syndrome, which remains elusive, and may highlight factors accounting for differences in medication response. The emerging role of genetic factors associated with steroid responsive and steroid-resistant forms of nephrotic syndrome within an ethnic group can provide insight into potential biological mechanisms leading to disease. For example, among African-Americans, the risk variants in *APOL1* are associated with a more than 10-fold increase in risk of focal segmental glomerulosclerosis and high-risk carriers have a twofold greater risk of progression to end-stage renal disease. Ongoing collaborative studies should consider capturing data on self-reported ethnicity to understand differences in incidence and outcomes. In the future, the availability of whole-genome data will provide an excellent opportunity for new clinical and translational research in childhood nephrotic syndrome and lead to a better understanding of the disease.

## Introduction

Nephrotic syndrome is a common childhood kidney disease characterized by a constellation of heavy proteinuria (urine protein to creatinine ratio >200 mg/mmol or ≥3 + proteinuria on urine dipstick) resulting in hypoalbuminemia (<25 g/L), hyperlipidemia, and peripheral edema (1). Despite treating nephrotic syndrome with prednisone for over 50 years, the exact mechanism of disease remains unclear. Most studies focus on podocyte injury (2), immunological (3), and environmental factors (4) as potential culprits in the pathogenesis of nephrotic syndrome, but causal factors remain elusive.

Corticosteroids remain the mainstay for treatment of nephrotic syndrome. Based on the response to corticosteroids, children with nephrotic syndrome segregate into a steroid-sensitive group that has a good long-term prognosis, but risk of frequent relapses, and a steroid-resistant group with higher risk of developing chronic kidney disease. Response to medications is quite variable with some children requiring further courses of steroid-sparing agents, while others achieve complete remission after the first course of prednisone. Steroid-sparing agents, such as cyclophosphamide, mycophenolate mofetil, calcineurin inhibitors, and rituximab, are often used to induce or maintain remission with mixed results. It is unclear what leads to this individual variability in drug response and the future risk of relapses.

Minimal change disease (MCD) is the most common histological variant of nephrotic syndrome and accounts for approximately 80% of cases in children based on historical data from 1967 to 1976 (5). Focal segmental glomerulosclerosis (FSGS) is less common but can be progressive with poor long-term outcomes. Data suggest differences in the incidence of FSGS among various ethnic groups, for example, a study involving 86 children from Kansas city, MO, USA, reported the annual incidence of FSGS of 1.6 per 100,000 in African-Americans compared to 0.3 per 100,000 in European Americans (6).

With recent advancements and improved understanding of the human genome, 45 genes are found to be associated with familial and sporadic nephrotic syndrome that primarily affect podocyte structure and function (7). Most recently identified, the gene *APOL1* is a major risk factor for FSGS among African-Americans accounting for progressive kidney disease. The influence of genetic factors among other ethnic groups is less clear.

This review focuses on the current understanding of ethnic differences in childhood nephrotic syndrome, discusses ongoing studies and role of ethnicity, and the influence of genetics within specific ethnic groups.

## Ethnic Differences in Nephrotic Syndrome

**Figure 1:**
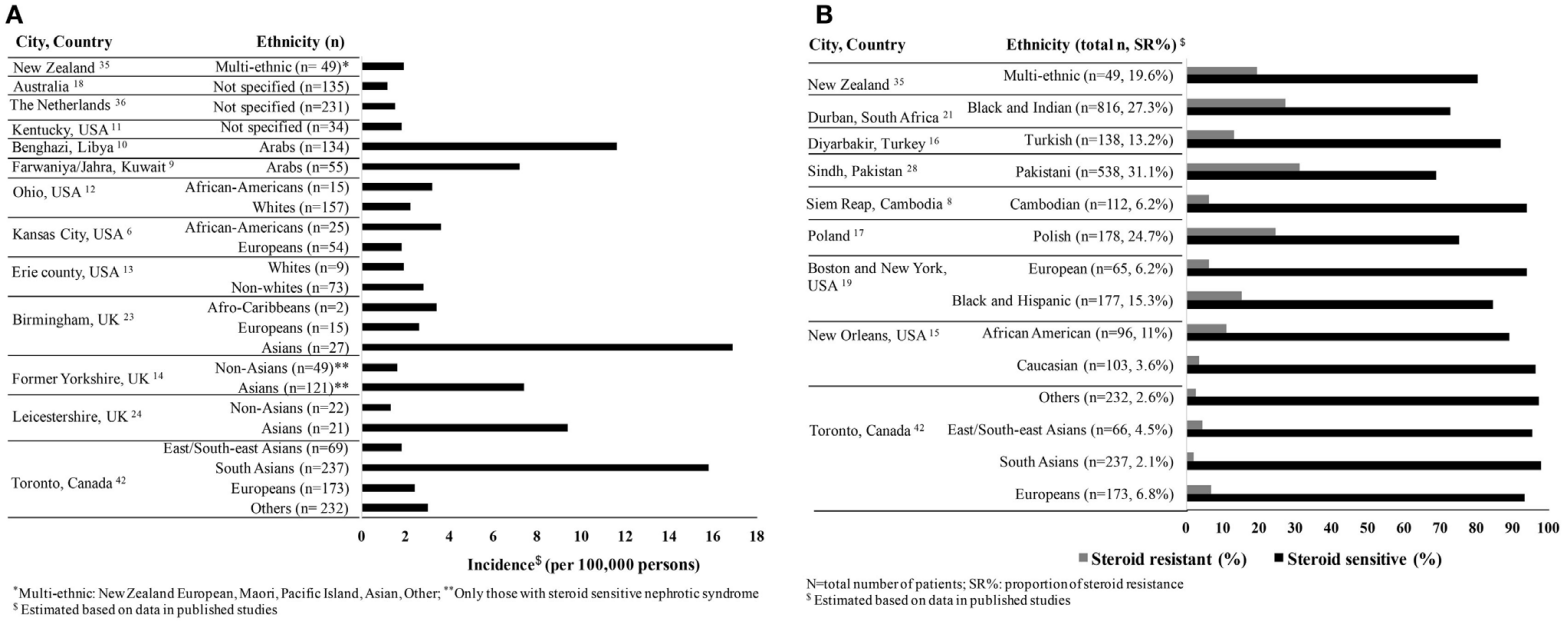
**(A)** Incidence of childhood nephrotic syndrome per 100,000 persons by ethnicity reported from 1946 to 2014. **(B)** Variability of steroid responsiveness by ethnicity among children with nephrotic syndrome in reported studies from 1986 to 2014.

Based on our review of the literature, the average incidence of nephrotic syndrome is 4.7 (range 1.15–16.9) per 100,000 persons in studies reported from 1946 to 2014, and the proportion with steroid resistance is 12.4% (range 2.1–27.3%) from 1986 to 2014 (Figures 1A,B). Hence, there is a considerable variation in disease burden by country of origin and steroid responsiveness, suggesting the potential role of ethnicity in susceptibility to disease (8–23).

In studies, where there are large diaspora populations, such as the United Kingdom, South Asians are reported to have a higher incidence of nephrotic syndrome ranging from 7.4 to 16.9 per 100,000 persons compared to Europeans (14, 24, 25). Studies from the US report a higher estimated incidence among children of African compared to European descent (Figure 1A) (6, 13). The proportion of steroid resistance also varies by ethnicity from 20% among Europeans, 16–27% among Africans, 27–54% among Asians, and 20–39% among South Asians (Figure 1B) (26–29). It is possible that these differences are at least partially attributable to variations in clinical management, selection bias, and definitions of outcomes. African-American children are more likely to have biopsy-proven FSGS (42–72%) with worse outcomes as compared to European children and commonly progress to end-stage renal disease (ESRD) (19, 26, 27, 30–32). Comparatively, the proportion of FSGS in India ranges from 15.3 to 39.1% (33, 34). All these reports have differing definitions, inconsistent inclusion, and selection criteria at tertiary centers and variable access to health care, which impact comparison of incidence rates and outcomes such as steroid responsiveness. In addition, most studies combine both steroid resistant and FSGS as a single category, thereby leading to further heterogeneity in study populations and impacting outcomes. On the other hand, registries from New Zealand and Netherlands did not show differences in disease burden by ethnicity primarily due to lack of power or reported ethnic diversity (35, 36). These registries or case series have short follow-up and limited clinical information; hence, differences in treatment response by ethnicity are not well reported. To address these limitations, a number of ongoing registries and cohort studies conducted worldwide deserve mention. These studies include children with various types of nephrotic syndrome with the overall goal to identify clinical, histological and genomic predictors of nephrotic syndrome. The highlights of each study are shown in Table 1.

**Table 1:** **Distribution of ethnic groups in ongoing glomerular disease registries and prospective studies**.

Study	Participating centers	Enrolled/projected participants	Initial start date	Inclusion criteria	Ethnic groups			
					Europeans (%)	South Asians (%)	East/South East Asians (%)	Others (%)
**Registries**								
PodoNet (37)	67	1655	2009	Congenital/steroid-resistant nephrotic syndrome	90.3	0.9	0.4	8.4^a^
RaDaR (38)	Not specified	220	2010	Children/adults with steroid-sensitive and -resistant nephrotic syndrome	76.6	2.6	0.06^d^	20^c^
**Cohort studies**								
NEPTUNE (39)	18	450	2010	Children/adults with MCD, FSGS, and MN	48	–	9	44^b^
INSIGHT (41, 42)	2	450	2012	All children with nephrotic syndrome	24	33	10	33^e^
CureGN	64	2400	2014	Children/adults with MCD, FSGS, IgA nephropathy, and MN	Not available			

*MCD, minimal change disease, FSGS, focal segmental glomerulosclerosis, MN, membranous nephropathy*.

*^a^Includes Hispanic, mixed, and Native Americans*.

*^b^Includes admixed Americans and Africans*.

*^c^Includes North African, East African, Afro-Caribbean, mixed, and unknown*.

*^d^Specified as “other Asian”*.

*^e^Includes individuals classified as West Central Asian/Middle Eastern, West Indian/Caribbean and African, Latin/Central/South American and Aboriginal, multi-ethnic, and unknown*.

PodoNet is an international registry that includes children with steroid-resistant disease and congenital nephrotic syndrome recruited predominantly from European countries having 90.3% participants of European descent (37). Registry for Rare Kidney Diseases (RaDaR), recently renamed the National Study for Nephrotic Syndrome (NephroS) study, is a web-based UK registry that aims to capture clinical information on all children and adults with nephrotic syndrome and studies to date have focused on genetic testing of steroid-resistant disease primarily involving Europeans (76.6%) (38).

Nephrotic Syndrome Study Network (NEPTUNE) and the Cure Glomerulonephropathy Network (CureGN) are both North American multicenter collaborative longitudinal cohort studies, which enroll both children and adults with biopsy-proven disease (MCD and FSGS). NEPTUNE recruits an incident cohort at the time of first biopsy and follows them closely for up to 5 years (39). It comprises a diverse ethnic cohort based on genetically derived ancestry. CureGN is a new multicenter study enrolling up to 2400 children and adults with incident or prevalent MCD and FSGS. All of these studies to date have limited enrollment of either South Asians or East/Southeast Asians as many may not receive a biopsy or fulfill entry criteria, thus limiting their generalizability to other patient populations.

Canadian Childhood Nephrotic Syndrome (CHILDNEPH) is enrolling children across Canada to study treatment strategies in nephrotic syndrome and system factors driving treatment variation (40). Insight into Nephrotic Syndrome: Investigating Genes, Health, and Therapeutics (INSIGHT) is an ongoing longitudinal study, initially from Toronto, ON, Canada, that aims to detect factors that affect disease susceptibility and treatment response among children with nephrotic syndrome (41). The preliminary results from this study (42) demonstrate that South Asians have approximately 6 times higher incidence of nephrotic syndrome but significantly lower odds of frequently relapsing disease as compared to Europeans.

There is a clear geographical variation in the incidence of nephrotic syndrome. It is possible that the differences in incidence by ethnicity are due to referral patterns at tertiary care centers or publication bias. The reason for variation in response to steroids and other immunosuppressive medications among specific ethnic groups has not been well documented. The results from registries and prospective studies will provide an excellent resource for future clinical and genetic research to understand disease pathogenesis and differences by ancestry.

A major limitation in understanding ethnic differences is the lack of detailed self-reported ethnicity data collected in most studies. Moreover, due to admixture it can be difficult to establish ethnicity among multi-ethnic children. Additionally, various cultural, environmental, and socioeconomic factors also act as important confounders and may influence individual variability to medication response and frequency of relapses. We provide a few suggestions for future studies in terms of collecting and reporting ethnicity data in children with nephrotic syndrome until genetic ancestry is available in some studies:

1. Ethnicity should be self-reported;
2. Ethnicity of two or more generations should be captured for multigenerational admixture;
3. A uniform definition of ethnic groups should be used to compare across studies;
4. Collaborative studies, both national and international, should include diverse ethnicities.

## Role of Ethnicity and Genetics in Childhood Nephrotic Syndrome

There is an evolving role of genetic risk and development of nephrotic syndrome in children. The discovery of *NPHS1* and *NPHS2* genes leading to congenital nephrotic syndrome provided the first evidence of a genetic cause of steroid-resistant disease. Since then, 45 genes have been associated with monogenic forms of nephrotic syndrome and highlight abnormalities in podocyte structure and function leading to disease (7). The current known genes associated with nephrotic syndrome account for only 20–30% of hereditary and 10–20% of sporadic cases. The majority of these genes especially *NPHS2* have been identified in European (43, 44), Japanese (45), and Turkish (46) families but have not been studied across broad ancestral groups where the allele frequency may vary and additional variants may be important. With advancement in genetic analyses, an increasing number of polymorphisms have been detected in children with nephrotic syndrome. It is important to note, however, that some variants are of unknown significance. It is difficult to determine whether these variants are common and/or pathogenic by ethnic or ancestral groups until genetic databases include more comprehensive information across many ethnic groups. Understanding the epidemiological differences in kidney disease by ethnicity may suggest possible genetic risk. For example, African-Americans are known to have a 7.5% lifetime risk of reaching end-stage kidney disease, which is significantly higher than approximately 2% in European Americans (47). In 2008, studies using mapping admixture by linkage disequilibrium identified loci on chromosome 22 that explained the higher incidence of development of FSGS and ESRD among Africans Americans compared to European Americans. Initially, genetic risk was attributed to variants in the *MYH9* gene (48, 49). With new data available in the HapMAP, the major source of genetic risk for African-American non-diabetic ESRD and FSGS was localized to *APOL1*, encoding apolipoprotein L1 (ApoL1), which is only 14 kb from the gene *MYH9* (50, 51). Risk of advanced kidney disease is two to seven times greater for those carrying risk alleles of *APOL1*, as compared to controls. The spectrum of *APOL1*-associated kidney disease is quite diverse and includes nephrotic syndrome (FSGS), non-diabetic chronic kidney disease secondary to hypertension, HIV-associated collapsing glomerulopathy (52), sickle cell nephropathy (53), and lupus nephritis (54). A recent study also demonstrated that the risk of progression of chronic kidney disease is approximately two times higher in African-Americans with *APOL1* variants despite adequate blood pressure control (55). Interestingly, some individuals carrying *APOL1* risk alleles do not develop kidney disease highlighting the role of additional genetic or environmental interactions (56). To further understand the association of *APOL1* variants with different rates of chronic kidney disease across various African countries and potential environmental interaction, the Kidney Disease Network of Human Hereditary and Health in Africa (H3Africa) consortium was established (57).

Recently, a study among 214 children with steroid-sensitive nephrotic syndrome from South Asia, specifically Sri Lanka, reported HLA-DQA1 missense coding variants as possible candidate loci based on exome array in case–control study compared to 149 healthy controls. The study findings were then confirmed among a replication sample of 100 European children with steroid-sensitive nephrotic syndrome and 205 European controls. This study supports the contributory role of immune response in the pathogenesis of nephrotic syndrome (58) and will need to be tested in additional cohorts with diverse ethnic groups. Previous smaller studies in Chinese and Japanese children have also reported association between variants in HLA-DQ3, HLA-DQ8, HLA-DR, HLADQW2, HLA-DQA1, and HLA-DQB1 in nephrotic syndrome (59–62).

## Ethnic Differences in Other Glomerular Diseases

In specific ethnic groups, genome-wide association studies have identified susceptibility loci for membranous and IgA nephropathies. HLA-DQA1 allele on chromosome 6p21 is most closely associated with idiopathic membranous nephropathy in persons of European ancestry (63). IgA nephropathy, the most common cause of glomerulonephritis in adults, has a higher prevalence in Asians as compared to North-Americans or Europeans (64). Recently, in a large diverse cohort of patients with IgA nephropathy, individuals with Pacific Asian origin were shown to have a higher risk of progression to ESRD (65). Studies among East Asians with IgA nephropathy have identified susceptibility loci with the strongest association in the region that include the HLA-DRB1, -DQA1, and -DQB1 genes (66). Similar studies are quite scarce in children with nephrotic syndrome.

## Conclusion

In children with nephrotic syndrome, incidence and response to treatment varies by ethnicity. It is likely that genetic and environmental risk factors play a substantial role in explaining these ethnic differences and need further study.

## Author Contributions

RC drafted the manuscript; RP conceptualized the work and critically revised the manuscript.

## Conflict of Interest Statement

The authors declare that the research was conducted in the absence of any commercial or financial relationships that could be construed as a potential conflict of interest.
